# (6-Meth­oxy-2-oxo-2*H*-chromen-4-yl)methyl morpholine-4-carbodithio­ate

**DOI:** 10.1107/S1600536812051847

**Published:** 2013-01-09

**Authors:** H. C. Devarajegowda, K. Mahesh Kumar, S. Seenivasa, H. K. Arunkashi, O. Kotresh

**Affiliations:** a Department of Physics, Yuvaraja’s College (Constituent College), University of Mysore, Mysore 570 005, Karnataka, India; b Department of Chemistry, Karnatak University’s Karnatak Science College, Dharwad, Karnataka 580 001, India; c Deapartment of Studies and Research in Chemistry, Tumkur University, Tumkur 572 103, Karnataka, India

## Abstract

In the title compound, C_16_H_17_NO_4_S_2_, the 2*H*-chromene ring system is nearly planar, with a maximum deviation of 0.070 (1) Å, and the morpholine ring adopts a chair conformation; the bond-angle sum for its N atom is 357.9°. The dihedral angle between the the 2*H*-chromene ring and the best plane through the morpholine ring is 89.09 (6)°. An intra­molecular C—H⋯S hydrogen bond occurs. In the crystal, C—H⋯O hydrogen bonds generate *R*_2_^2^(8) rings and π–π inter­actions occur between fused benzene rings of the chromene system [shortest centroid–centroid distance = 3.5487 (8) Å].

## Related literature

For a related structure, background to coumarins and details of the synthesis of the title compound, see: Kumar *et al.* (2012 ▶).
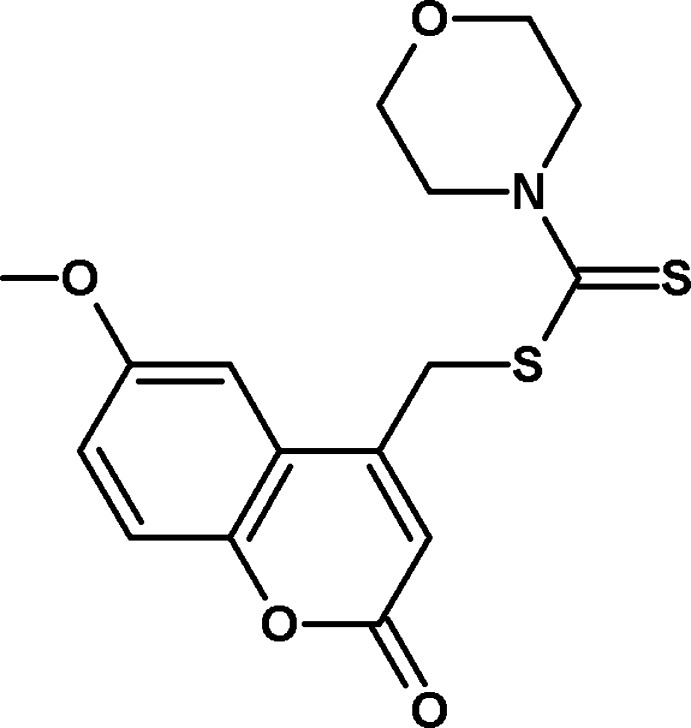


## Experimental

### 

#### Crystal data

C_16_H_17_NO_4_S_2_
*M* *_r_* = 351.43
Triclinic, 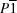

*a* = 7.0026 (5) Å
*b* = 7.9939 (6) Å
*c* = 14.8033 (11) Å
α = 75.433 (4)°
β = 86.642 (4)°
γ = 78.355 (4)°
*V* = 785.49 (10) Å^3^
*Z* = 2
Mo *K*α radiation
μ = 0.36 mm^−1^
*T* = 296 K
0.24 × 0.20 × 0.12 mm

#### Data collection

Bruker SMART CCD area-detector diffractometer
Absorption correction: multi-scan (*SADABS*; Sheldrick, 2007 ▶) *T*_min_ = 0.770, *T*_max_ = 1.000
13583 measured reflections
2725 independent reflections
2482 reflections with *I* > 2σ(*I*)
*R*_int_ = 0.024

#### Refinement

*R*[*F* ^2^ > 2σ(*F* ^2^)] = 0.027
*wR*(*F* ^2^) = 0.073
*S* = 1.06
2725 reflections
208 parameters
H-atom parameters constrained
Δρ_max_ = 0.21 e Å^−3^
Δρ_min_ = −0.15 e Å^−3^

### 

Data collection: *SMART* (Bruker, 2001 ▶); cell refinement: *SAINT* (Bruker, 2001 ▶); data reduction: *SAINT*; program(s) used to solve structure: *SHELXS97* (Sheldrick, 2008 ▶); program(s) used to refine structure: *SHELXL97* (Sheldrick, 2008 ▶); molecular graphics: *ORTEP-3 for Windows* (Farrugia, 2012 ▶); software used to prepare material for publication: *SHELXL97*.

## Supplementary Material

Crystal structure: contains datablock(s) I, global. DOI: 10.1107/S1600536812051847/gw2129sup1.cif

Structure factors: contains datablock(s) I. DOI: 10.1107/S1600536812051847/gw2129Isup2.hkl

Supplementary material file. DOI: 10.1107/S1600536812051847/gw2129Isup3.cml

Additional supplementary materials:  crystallographic information; 3D view; checkCIF report

## Figures and Tables

**Table 1:** Hydrogen-bond geometry (Å, °)

*D*—H⋯*A*	*D*—H	H⋯*A*	*D*⋯*A*	*D*—H⋯*A*
C14—H14⋯O6^i^	0.93	2.55	3.4582 (19)	166
C17—H17*B*⋯O3^ii^	0.96	2.57	3.386 (2)	143
C18—H18*B*⋯S2	0.97	2.55	3.1527 (14)	120

Symmetry codes: (i) 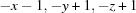; (ii) 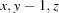.

**Fig. 1.**
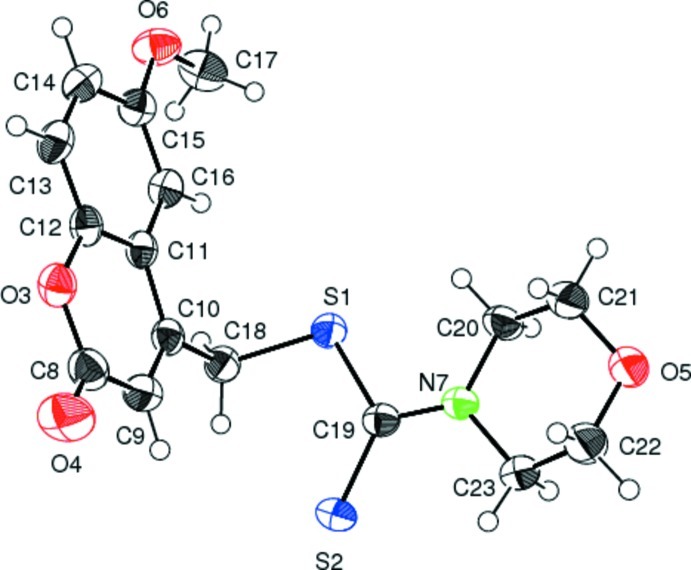
The molecular structure of the title compound. Displacement ellipsoids are drawn at the 50% probability level. Hydrogen atoms are shown as spheres of arbitrary radius.

**Fig. 2.**
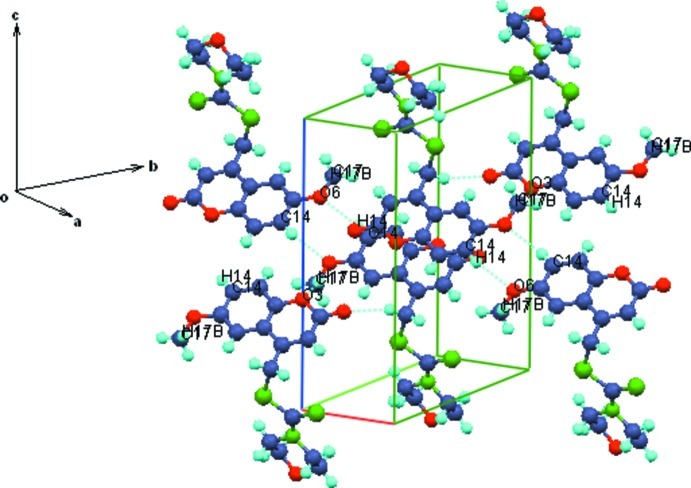
The packing of molecules.

## supplementary crystallographic information

### Comment

As part of our ongoing studies of coumarins (or 2*H*-chromen-2-ones) with possible biological activities (Kumar *et al.*, 2012), we now describe the structure of (6-methoxy-2-oxo-2*H*-chromen-4-yl) methyl morpholine-4-carbodithioate.

The asymmetric unit of (6-methoxy-2-oxo-2*H*-chromen-4-yl)methyl morpholine-4-carbodithioate is shown in Fig. 1. The 2*H*-chromene ring system (O3/C8–C16) is essentially planar, with a maximum deviation of 0.070 (1) Å for atom C8 and the morpholine ring adopts a chair conformation: the bond-angle sum for its N7 atom is 357.9 Å. The dihedral angle between the 2*H*-chromene (O3/C8–C16) ring and the morpholine (N7/O5/C20–C23) ring is 89.09 (6)°. In the crystal structure, (Fig. 2), intermolecular C14—H14···O6 and C17B—H17B···O3 and intramolecular C18—H18B···S2 hydrogen bonds observed and also π–π interactions between fused benzene *Cg*_(3)_ (C11–C16) rings of chromene [shortest centroid–centroid distance = 3.5487 (8) Å] further stabilize the crystal packing

### Experimental

This compound was prepared according to the reported method (Kumar *et al.*, 2012). Colourless needles of the title compound were grown from a mixed solution of EtOH / CHCl_3_(V/V = 1/1) by slow evaporation at room temperature. Colour: yellowish. Yield= 84%, m.p.481 K.

### Refinement

All H atoms were positioned geometrically, with C—H = 0.93 Å for aromatic H, C—H = 0.97 Å for methylene H and C—H = 0.96 Å for methyl H,and refined using a riding model with *U*_iso_(H) = 1.5*U*_eq_(C) for methyl H and *U*_iso_(H) = 1.2*U*_eq_(C) for all other H.

### Crystal data

**Table:**

C_16_H_17_NO_4_S_2_	*Z* = 2
*M**_r_* = 351.43	*F*(000) = 368
Triclinic, *P*1	*D*_x_ = 1.486 Mg m^−^^3^
Hall symbol: -P 1	Melting point: 481 K
*a* = 7.0026 (5) Å	Mo *K*α radiation, λ = 0.71073 Å
*b* = 7.9939 (6) Å	Cell parameters from 2725 reflections
*c* = 14.8033 (11) Å	θ = 2.7–25.0°
α = 75.433 (4)°	µ = 0.36 mm^−^^1^
β = 86.642 (4)°	*T* = 296 K
γ = 78.355 (4)°	Plate, colourless
*V* = 785.49 (10) Å^3^	0.24 × 0.20 × 0.12 mm

### Data collection

**Table:**

Bruker SMART CCD area-detector diffractometer	2725 independent reflections
Radiation source: fine-focus sealed tube	2482 reflections with *I* > 2σ(*I*)
Graphite monochromator	*R*_int_ = 0.024
ω and φ scans	θ_max_ = 25.0°, θ_min_ = 2.7°
Absorption correction: multi-scan (*SADABS*; Sheldrick, 2007)	*h* = −8→8
*T*_min_ = 0.770, *T*_max_ = 1.000	*k* = −9→9
13583 measured reflections	*l* = −17→17

### Refinement

**Table:**

Refinement on *F*^2^	Primary atom site location: structure-invariant direct methods
Least-squares matrix: full	Secondary atom site location: difference Fourier map
*R*[*F*^2^ > 2σ(*F*^2^)] = 0.027	Hydrogen site location: inferred from neighbouring sites
*wR*(*F*^2^) = 0.073	H-atom parameters constrained
*S* = 1.06	*w* = 1/[σ^2^(*F*_o_^2^) + (0.0403*P*)^2^ + 0.1479*P*] where *P* = (*F*_o_^2^ + 2*F*_c_^2^)/3
2725 reflections	(Δ/σ)_max_ = 0.001
208 parameters	Δρ_max_ = 0.21 e Å^−^^3^
0 restraints	Δρ_min_ = −0.15 e Å^−^^3^

### Special details

**Table:**

Experimental. IR (KBr): 662 cm^-1^(C—S), 1233 cm^-1^1 (C=S), 1032 cm^-1^(C—O), 842 cm^-1^ (C—N),1118 cm^-1^(C—O—C), 1703 cm^-1^(C=O). GCMS: m/e: 335. 1H NMR (400 MHz, CDCl_3_, \?, p.p.m.) 1.91 (m, 6H, Morpholine-CH_2_), 2.34 (s, 4H, Morpholine –CH_2_), 4.63 (d, 2H, Methylene-CH_2_),5.88(s, 1H, Ar—H), 6.39 (s, 1H, Ar—H), 7.08 (s, 1H, Ar—H), 7.12 (s, 1H, Ar—H). Elemental analysis for C_16_H_17_NO_3_S_2_: C, 57.21; H, 5.04; N, 4.11.
Geometry. All s.u.'s (except the s.u. in the dihedral angle between two l.s. planes) are estimated using the full covariance matrix. The cell s.u.'s are taken into account individually in the estimation of s.u.'s in distances, angles and torsion angles; correlations between s.u.'s in cell parameters are only used when they are defined by crystal symmetry. An approximate (isotropic) treatment of cell s.u.'s is used for estimating s.u.'s involving l.s. planes.
Refinement. Refinement of *F*^2^ against ALL reflections. The weighted *R*-factor *wR* and goodness of fit *S* are based on *F*^2^, conventional *R*-factors *R* are based on *F*, with *F* set to zero for negative *F*^2^. The threshold expression of *F*^2^ > 2σ(*F*^2^) is used only for calculating *R*-factors(gt) *etc*. and is not relevant to the choice of reflections for refinement. *R*-factors based on *F*^2^ are statistically about twice as large as those based on *F*, and *R*- factors based on ALL data will be even larger.

### Fractional atomic coordinates and isotropic or equivalent isotropic displacement parameters (Å^2^)

**Table:**

	*x*	*y*	*z*	*U*_iso_*/*U*_eq_	
S1	0.20243 (5)	0.57411 (5)	0.13634 (2)	0.03769 (12)	
S2	0.56769 (5)	0.71727 (6)	0.06898 (3)	0.04523 (13)	
O3	0.11086 (15)	0.92127 (12)	0.39849 (7)	0.0387 (2)	
O4	0.3416 (2)	1.06890 (16)	0.34092 (10)	0.0637 (3)	
O5	0.05206 (16)	0.92565 (15)	−0.20255 (7)	0.0481 (3)	
O6	−0.32490 (16)	0.41563 (13)	0.41094 (8)	0.0478 (3)	
N7	0.25269 (16)	0.75947 (14)	−0.03248 (8)	0.0338 (3)	
C8	0.2732 (2)	0.94051 (19)	0.34374 (10)	0.0423 (3)	
C9	0.3469 (2)	0.80463 (19)	0.29565 (10)	0.0391 (3)	
H9	0.4639	0.8085	0.2627	0.047*	
C10	0.2545 (2)	0.67253 (17)	0.29619 (9)	0.0316 (3)	
C11	0.0733 (2)	0.66496 (16)	0.34768 (8)	0.0302 (3)	
C12	0.0084 (2)	0.79116 (16)	0.39807 (9)	0.0322 (3)	
C13	−0.1612 (2)	0.79099 (18)	0.45089 (9)	0.0380 (3)	
H13	−0.2017	0.8758	0.4846	0.046*	
C14	−0.2691 (2)	0.66436 (19)	0.45309 (10)	0.0396 (3)	
H14	−0.3836	0.6639	0.4882	0.047*	
C15	−0.2080 (2)	0.53636 (17)	0.40304 (9)	0.0355 (3)	
C16	−0.0384 (2)	0.53634 (17)	0.35082 (9)	0.0336 (3)	
H16	0.0020	0.4508	0.3176	0.040*	
C17	−0.2599 (2)	0.2748 (2)	0.36796 (13)	0.0518 (4)	
H17A	−0.3539	0.1994	0.3787	0.078*	
H17B	−0.1370	0.2086	0.3939	0.078*	
H17C	−0.2448	0.3208	0.3020	0.078*	
C18	0.3358 (2)	0.53685 (18)	0.24300 (9)	0.0353 (3)	
H18A	0.3293	0.4206	0.2818	0.042*	
H18B	0.4718	0.5408	0.2280	0.042*	
C19	0.34447 (19)	0.69401 (16)	0.04920 (9)	0.0311 (3)	
C20	0.0686 (2)	0.71676 (19)	−0.05329 (10)	0.0407 (3)	
H20A	−0.0043	0.6857	0.0042	0.049*	
H20B	0.0962	0.6156	−0.0802	0.049*	
C21	−0.0524 (2)	0.8689 (2)	−0.11983 (11)	0.0441 (4)	
H21A	−0.1690	0.8341	−0.1351	0.053*	
H21B	−0.0923	0.9658	−0.0901	0.053*	
C22	0.2181 (2)	0.9831 (2)	−0.18091 (11)	0.0497 (4)	
H22A	0.1762	1.0806	−0.1518	0.060*	
H22B	0.2864	1.0260	−0.2383	0.060*	
C23	0.3561 (2)	0.8398 (2)	−0.11672 (10)	0.0430 (3)	
H23A	0.4142	0.7502	−0.1491	0.052*	
H23B	0.4600	0.8887	−0.0989	0.052*	

### Atomic displacement parameters (Å^2^)

**Table:**

	*U*^11^	*U*^22^	*U*^33^	*U*^12^	*U*^13^	*U*^23^
S1	0.0381 (2)	0.0462 (2)	0.0334 (2)	−0.01826 (16)	0.00258 (14)	−0.01081 (15)
S2	0.0298 (2)	0.0622 (3)	0.0451 (2)	−0.01602 (17)	−0.00144 (16)	−0.00997 (18)
O3	0.0470 (6)	0.0347 (5)	0.0383 (5)	−0.0103 (4)	−0.0010 (4)	−0.0142 (4)
O4	0.0665 (8)	0.0560 (7)	0.0859 (9)	−0.0322 (6)	0.0105 (7)	−0.0352 (6)
O5	0.0433 (6)	0.0585 (7)	0.0405 (6)	−0.0172 (5)	−0.0066 (5)	−0.0014 (5)
O6	0.0432 (6)	0.0412 (6)	0.0627 (7)	−0.0158 (5)	0.0115 (5)	−0.0164 (5)
N7	0.0285 (6)	0.0383 (6)	0.0351 (6)	−0.0111 (5)	0.0005 (5)	−0.0066 (5)
C8	0.0440 (9)	0.0426 (8)	0.0438 (8)	−0.0140 (7)	−0.0039 (7)	−0.0118 (6)
C9	0.0347 (8)	0.0430 (8)	0.0414 (8)	−0.0098 (6)	0.0002 (6)	−0.0118 (6)
C10	0.0328 (7)	0.0316 (6)	0.0278 (6)	−0.0024 (5)	−0.0047 (5)	−0.0045 (5)
C11	0.0343 (7)	0.0283 (6)	0.0254 (6)	−0.0030 (5)	−0.0039 (5)	−0.0032 (5)
C12	0.0397 (8)	0.0283 (6)	0.0274 (6)	−0.0052 (6)	−0.0052 (6)	−0.0044 (5)
C13	0.0457 (9)	0.0344 (7)	0.0323 (7)	−0.0020 (6)	0.0026 (6)	−0.0107 (6)
C14	0.0400 (8)	0.0397 (7)	0.0356 (7)	−0.0049 (6)	0.0065 (6)	−0.0067 (6)
C15	0.0372 (8)	0.0307 (7)	0.0361 (7)	−0.0076 (6)	−0.0006 (6)	−0.0029 (5)
C16	0.0397 (8)	0.0279 (6)	0.0331 (7)	−0.0042 (6)	0.0001 (6)	−0.0090 (5)
C17	0.0464 (9)	0.0387 (8)	0.0733 (11)	−0.0102 (7)	−0.0034 (8)	−0.0174 (8)
C18	0.0342 (7)	0.0351 (7)	0.0344 (7)	−0.0033 (6)	−0.0012 (6)	−0.0072 (5)
C19	0.0292 (7)	0.0295 (6)	0.0366 (7)	−0.0051 (5)	0.0034 (5)	−0.0127 (5)
C20	0.0337 (8)	0.0456 (8)	0.0436 (8)	−0.0170 (6)	−0.0039 (6)	−0.0041 (6)
C21	0.0327 (8)	0.0516 (9)	0.0454 (8)	−0.0084 (7)	−0.0024 (6)	−0.0068 (7)
C22	0.0481 (10)	0.0533 (9)	0.0456 (9)	−0.0225 (8)	−0.0038 (7)	0.0023 (7)
C23	0.0342 (8)	0.0559 (9)	0.0381 (8)	−0.0149 (7)	0.0036 (6)	−0.0061 (7)

### Geometric parameters (Å, º)

**Table:**

S1—C19	1.7846 (13)	C13—C14	1.373 (2)
S1—C18	1.8107 (14)	C13—H13	0.9300
S2—C19	1.6620 (14)	C14—C15	1.395 (2)
O3—C8	1.3682 (18)	C14—H14	0.9300
O3—C12	1.3792 (16)	C15—C16	1.378 (2)
O4—C8	1.2083 (18)	C16—H16	0.9300
O5—C21	1.4105 (19)	C17—H17A	0.9600
O5—C22	1.4136 (19)	C17—H17B	0.9600
O6—C15	1.3661 (17)	C17—H17C	0.9600
O6—C17	1.4134 (19)	C18—H18A	0.9700
N7—C19	1.3363 (17)	C18—H18B	0.9700
N7—C20	1.4662 (18)	C20—C21	1.499 (2)
N7—C23	1.4733 (18)	C20—H20A	0.9700
C8—C9	1.440 (2)	C20—H20B	0.9700
C9—C10	1.344 (2)	C21—H21A	0.9700
C9—H9	0.9300	C21—H21B	0.9700
C10—C11	1.4453 (19)	C22—C23	1.504 (2)
C10—C18	1.4995 (18)	C22—H22A	0.9700
C11—C12	1.3909 (19)	C22—H22B	0.9700
C11—C16	1.4028 (19)	C23—H23A	0.9700
C12—C13	1.383 (2)	C23—H23B	0.9700
			
C19—S1—C18	103.85 (6)	O6—C17—H17C	109.5
C8—O3—C12	121.50 (11)	H17A—C17—H17C	109.5
C21—O5—C22	109.50 (12)	H17B—C17—H17C	109.5
C15—O6—C17	117.51 (12)	C10—C18—S1	111.36 (9)
C19—N7—C20	124.03 (11)	C10—C18—H18A	109.4
C19—N7—C23	120.79 (12)	S1—C18—H18A	109.4
C20—N7—C23	113.08 (11)	C10—C18—H18B	109.4
O4—C8—O3	117.00 (14)	S1—C18—H18B	109.4
O4—C8—C9	126.27 (15)	H18A—C18—H18B	108.0
O3—C8—C9	116.73 (12)	N7—C19—S2	124.57 (10)
C10—C9—C8	122.97 (14)	N7—C19—S1	112.68 (10)
C10—C9—H9	118.5	S2—C19—S1	122.74 (8)
C8—C9—H9	118.5	N7—C20—C21	111.40 (12)
C9—C10—C11	118.87 (13)	N7—C20—H20A	109.3
C9—C10—C18	120.67 (13)	C21—C20—H20A	109.3
C11—C10—C18	120.46 (12)	N7—C20—H20B	109.3
C12—C11—C16	118.41 (13)	C21—C20—H20B	109.3
C12—C11—C10	117.82 (12)	H20A—C20—H20B	108.0
C16—C11—C10	123.77 (12)	O5—C21—C20	111.41 (12)
O3—C12—C13	116.84 (12)	O5—C21—H21A	109.3
O3—C12—C11	121.63 (12)	C20—C21—H21A	109.3
C13—C12—C11	121.52 (13)	O5—C21—H21B	109.3
C14—C13—C12	119.35 (13)	C20—C21—H21B	109.3
C14—C13—H13	120.3	H21A—C21—H21B	108.0
C12—C13—H13	120.3	O5—C22—C23	112.76 (13)
C13—C14—C15	120.46 (13)	O5—C22—H22A	109.0
C13—C14—H14	119.8	C23—C22—H22A	109.0
C15—C14—H14	119.8	O5—C22—H22B	109.0
O6—C15—C16	124.29 (13)	C23—C22—H22B	109.0
O6—C15—C14	115.64 (13)	H22A—C22—H22B	107.8
C16—C15—C14	120.07 (13)	N7—C23—C22	110.63 (12)
C15—C16—C11	120.20 (12)	N7—C23—H23A	109.5
C15—C16—H16	119.9	C22—C23—H23A	109.5
C11—C16—H16	119.9	N7—C23—H23B	109.5
O6—C17—H17A	109.5	C22—C23—H23B	109.5
O6—C17—H17B	109.5	H23A—C23—H23B	108.1
H17A—C17—H17B	109.5		

### Hydrogen-bond geometry (Å, º)

**Table:**

*D*—H···*A*	*D*—H	H···*A*	*D*···*A*	*D*—H···*A*
C14—H14···O6^i^	0.93	2.55	3.4582 (19)	166
C17—H17*B*···O3^ii^	0.96	2.57	3.386 (2)	143
C18—H18*B*···S2	0.97	2.55	3.1527 (14)	120

Symmetry codes: (i) −*x*−1, −*y*+1, −*z*+1; (ii) *x*, *y*−1, *z*.
